# Impact Measurement of COVID-19 Lockdown on China’s Electricity-Carbon Nexus

**DOI:** 10.3390/ijerph18189736

**Published:** 2021-09-15

**Authors:** Mingyue Zhao, Yuqing Niu, Lei Tian, Yizhi Liu, Qiang Zhai

**Affiliations:** 1 Department of Mechanical Engineering, School of Mechanical, Electrical & Information Engineering, Shandong University (Weihai), Weihai 264209, China; myzhao@mail.sdu.edu.cn (M.Z.); yuqingniu@mail.sdu.edu.cn (Y.N.); 201700800297@mail.sdu.edu.cn (Y.L.); 2 Medical Examination Center, Peking University Third Hospital, Beijing 100191, China

**Keywords:** COVID-19, lockdown, electricity, carbon, China

## Abstract

Lockdown measures to prevent the spread of coronavirus disease 2019 (COVID-19) resulted in the plummeting of China’s overall electric-power demand and production. To date, power generation remains one of the largest carbon dioxide (CO_2_) emitting sectors of China on account of its high carbon intensity. Within this context, our study seeks to measure the impacts of COVID-19 lockdown on the electricity-power related carbon footprints on both generation and consumption sides. Built on statistical data of electricity generation and consumption released by the National Bureau of Statistics of China (NBSC), we calculate he nationwide electricity related CO_2_ emission changes in regional, economic-sectoral and technological dimensions during January–April 2020, when the strictest lock-down measures were taken in China and compare the results with the same months of the year prior. Our results show that both east and central China power grids witnessed drastic reduction (15.0% and 13.8%) in electricity-generation caused CO_2_ emissions; and the biggest falls of provincial-scale electricity-generation CO_2_ emission took place in Hubei (27.3%). Among China’s electricity production mix, coal remains the biggest CO_2_ emitter and contributed 95.7% of the overall nationwide reduction. The most significant decline of the nationwide consumptive-electricity carbon footprint was by 10.1% in February, with the secondary economic sector the biggest contributor.

## 1. Introduction

The outbreak of coronavirus disease 2019 (COVID-19) has significantly damaged the global economy and public health. On the other hand, the subsequent prevention and control measures offer us an unprecedented opportunity to investigate the impacts of large scale social and economic behavior changes on overall environmental qualities [1]. Given that measurement and monitoring are among the 10 essential services of environmental public health [2], many efforts have been devoted to studying the impacts of COVID-19 lockdown policies on the environment and public health from different perspectives, including the measurement of atmospheric carbon dioxide (CO_2_) concentration changes. A recent review by Sharifi et al. reviewed the impacts of COVID-19 from different perspectives, such as environmental quality, socio-economic impacts, management and governance, transportation and urban design, as well as overarching issues [3]. Berman et al. reported a 25.5% reduction of NO_2_ in the US during 13 March to 21 April comparted to the pre-COVID-19 period (8 January to 12 March) [4]. Baldasano’s study shows that the reductions of NO_2_ concentrations in Spain’s two largest cities were 62% and 50% [5]. Bao et al. studied the air qualities in 44 cities in northern China and found the concentrations of SO_2_, PM_2.5_, PM_10_, NO_2_ and CO decreased by 6.76%, 5.93%, 13.66%, 24.67% and 4.58% [6].

Studies show that the pandemic and its associated prevention and control measures, i.e., lockdown and stay-home policies, temporarily improve the global air qualities in terms of short-term declines of airborne nitrogen dioxide (NO_2_) and CO_2_ emissions [7], as the lockdown resulted in a considerable slowing of human activities and power demand [7,8,9,10,11,12,13,14], contributing to the decline associated with power sector emissions [9,15,16,17].

Reported studies with respect to COVID-19 lockdown’s environmental impacts are mostly based either on statistical data, i.e., bottom-up method, or on satellite-observed Nitrogen Dioxide retrieval data, i.e., top-down method [18]. Taking the case of China, both local and national CO_2_ concentration reduction has been estimated through either method. An example of the bottom-up method application is the estimation of CO_2_ emission reductions in the first quarter (Q1) of 2020, using national and provincial GDP data as well as the China emission accounts and datasets (CEADs) inventory [19]. Top-down methods was applied for both regional- and national-scale studies; for instance, the reduction of atmospheric CO_2_ concentration on East China was estimated using space-based observations, via analyzing a small ensemble of OCO-2 and GOSAT satellite retrievals of column averaged dry-air mole fractions of CO_2_, i.e., XCO_2_ [20]. However, the analysis therein pointed out that it is challenging to reliably detect and accurately quantify the emission reduction with current satellite data sets. TROPOMI observation of NO_2_ was used to deduce 10-day moving averages of NOx and CO_2_ emissions over China, differentiating emissions by sectors and provinces, and the results demonstrated an 11.5% reduction of China’s CO_2_ emission between January–April 2020 compared to the same period in 2019 [21]. The authors further discussed the uncertainties and limitations for each step of their analysis. In a recent study, results were reported involving both statistical data for energy consumption and satellite retrievals of NO_2_ column concentration for estimating the correlation between rate of daily new COVID-19 cases and constrained-activity related CO_2_ emissions therein. Aside from the above bottom-up and top-down methods, ground observation was adopted as well to assess the impact of COVID-19 lockdown on atmospheric CO_2_ concentration in Xi’an city, Shanxi, through δ13C measurement with weather influence corrections [22]. Likewise, another recent study presented its estimation of fossil fuel CO_2_ reduction in China during COVID-19 via ground observation of the outflow of emissions from China [23]. 

Our literature review reveals the following research gaps and limitations as:

(1) as of writing, limited studies reported on the COVID-19-caused CO_2_ reductions for electric-power sector, as most of the studies focus on the overall airborne CO_2_ emissions or concentration and lack a decomposition or breakdown view;
(2) current existing studies are mostly based on numerically predicted data without official validation which damages the reliability of their findings;
(3) presented uncertainty analyses are conducted more as qualitative analyses and more in a qualitative manner thus lack adequate quantitative identification.

To address the gaps, this study builds upon the government-released statistical data of China’s electricity generation and consumption between January and April 2020, in which the strictest lock-down measures were taken in China, and explores from multiple perspectives and dimensions, i.e., generation and consumption, nationwide and provincial scales, as well as various industrial-sector-wise. Specifically, this study is conducted with trifold aims:

(1) to perform an in-depth and extensive measurement of monthly carbon footprint changes of China’s electric-power generation and consumption in various dimensions to increase comprehending of their relationships with the lockdown measures;
(2) to investigate the COVID-19 impact on China’s electricity-carbon nexus based on the official released statistical data, to avoid the unreliability from predictions and assumptions;
(3) to integrate Monte-Carlo method to the systematic approach to quantitatively test the uncertainty propagation effects and the probability distributions of the results, thereby to improve the reliability and confidence level of the measurement and diagnostics;
(4) last but not least, to identify issues of China’s official released statistical data in power sector.

The results of this study could provide more insights into the COVID-19 effect on China’s electricity-carbon nexus and raise attention to the average public and decision makers in charge of the overall China’s environmental management and sustainability improvement, especially under the ambitious goals of peaking its carbon emissions before 2030 and neutralizing its carbon emissions before 2060 [24].

## 2. Materials and Methods

On 23 January 2020, Wuhan, the capital of Hubei Province, China declared the shutdown of transportation, cancellation of domestic film releasement, followed by the implementation of provincial First-Level Public Health Emergency Response (FLPHER) the same day. On 24 January, the Chinese Spring Festival, subsequent measures were declared in succession such as cinema shutdown, tourists’ sites shutdown, inter-provincial buses and trains shutdown, closure of schools, restrictions on groups and communities, as well as requirements for residents not to return from holiday for 14 days, etc. These first level emergency measures were downgraded to second level emergency on 2 May 2020.

The CO_2_ emissions for electricity generation and consumption are calculated separately. For the electricity generation, first, the CO_2_ intensities for each power generation technology are identified, multiplying the generation amount to get the total CO_2_ amount related to the generations from each technology. Then, the total CO_2_ from all power generation technologies is obtained by summing them up. Further, on the consumption side, the CO_2_ burdens from purchased electricity are complex, as the electricity is normally from various power girds, so that the provincial CO_2_ emissions from electricity consumptions are calculated by multiplying the provincial consumption amounts with the CO_2_ burdens of the grids to which those provinces belong. The method is explained in detail in the following sections.

### 2.1. Estimation of Monthly CO_2_ Emissions from Electric-Power Generation

China National Bureau of Statistics released the monthly electric-power generation data of 2019 and 2020, including data of coal, hydro, wind, nuclear and solar data for 31 provinces of Mainland China. We took the carbon intensities of each individual power generation technology, multiplied their associated power generation and summed them up to calculate the sub-total CO_2_ emissions of each province and the national total, as presented below:

(1) $$E_{i}=\sum_{j=1}^{31}\sum_{k=1}^{5}I_{k}\times G_{i,j,k}\;$$

(2) $$E_{k}=\sum_{i=1}^{4}\sum_{j=1}^{31}I_{k}\times G_{i,j,k}\;$$

(3) $$E_{Total}=\sum_{i=1}^{4}\sum_{j=1}^{31}\sum_{k=1}^{5}I_{k}\times G_{i,j,k}\;$$

where $E_{Total}$ is the total national CO_2_ emissions from electric-power generation from January to April 2020; $E_{i}$ is the total CO_2_ emissions by each technology in the *i*th month; $E_{k}$ is the monthly total emissions of the *k*th technology; *i* stands for the index of each month; *j* stands for the index of each of 31 provinces; *k* is the index of each of the 5 power generation technologies; namely, coal, hydro, wind, nuclear and solar. $G_{i,j,k}$ is the electricity generation with the *k*th technology in the *i*th month in the *j*th province.

### 2.2. Estimation of Monthly Consumptive-Electricity Related CO_2_ Emissions

There are six national power grids in China, including the Northeast, Northwest, North, Central, East and South China Power Grids [25,26]. As shown in Table 1, the geographical coverages of these grids are as such: Northeast China Grid (NCG) covers Liaoning, Jilin, Heilongjiang, East Inner Mongolia; Northwest China Grid (NWCG) covers Gansu, Qinghai, Ningxia, Xinjiang, Tibet and Shanxi; North China Grid (NCG) covers Beijing, Tianjin, Hebei, Shandong, Shanxi and Western Inner Mongolia; Central China Grid (CCG) covers Hubei, Hunan, Jiangxi, Sichuan, Chongqing and Henan; East China Grid (ECG) covers Zhejiang, Jiangsu, Shanghai, Anhui, Fujian; South China Grid (SCG) covers Guangdong, Guangxi, Guizhou, Yunnan, Hainan.

Having identified which power grid each province belongs to, we took the latest carbon intensity values of the six national grids [27], as shown in Table 2, and calculated the total four-month carbon footprint for each province and the national total with the following equations:

(4) $$E_{i,cons.}=\sum_{j=1}^{31}\left(CI_{l}\times C_{i,j,l}\right)\;$$

(5) $$E_{total,cons.}=\sum_{i=1}^{4}\sum_{j=1}^{31}\left(CI_{l}\times C_{i,j,l}\right)\;$$

where $E_{i,cons.}$ is the consumptive-electricity related carbon emissions in the *i*th month; $E_{total,cons.}$ is the total four month consumptive-electricity carbon footprint in Mainland China; *i* is the index of each month between January to April 2020; *j* stands for the index of each of 31 provinces; *l* stands for the index of each power grid; $CI_{l}$ is the carbon intensity of the *l*th national power grid; $C_{i,j,l}$ is the electricity consumption of the *j*th province during the *i*th month.

### 2.3. A Case Study of Hubei Province

#### 2.3.1. Statistics of Hubei Monthly Electricity Consumption

Hubei is the most affected province by COVID-19 in China, and thus we investigate the monthly changes of electric power consumption of three main Hubei-provincial economic sectors, i.e., primary, secondary and tertiary sectors, as well as urban- and rural-residential electricity usage, alongside implementations of the COVID-19 prevention and control measures from January through April 2020. We conduct this investigation to increase understanding of the associations between timings of the transmission prevention and control measures and the changes of the sectoral electricity consumptions. The data of the same period in 2019 is used for comparison as the baseline to discover the temporal variations of the monthly electric power usage.

#### 2.3.2. Calculation of Hubei Monthly Consumptive-Electricity Carbon Footprint

**Figure 1:**
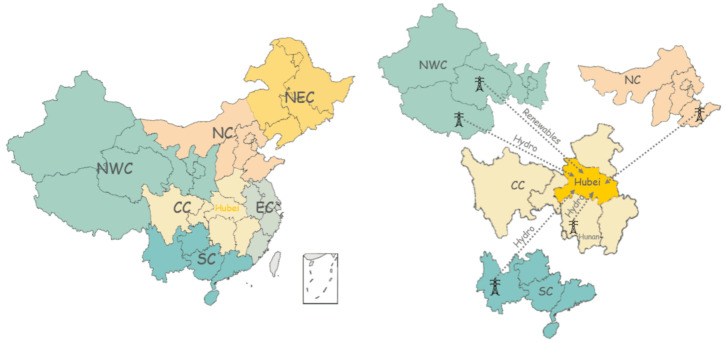
Trans-regional electricity purchase in Hubei in 2017. Data from [25]. Note: Northwest China (NWC), Central China (CC), East China (EC), Northeast China (NEC), South China (SC), North China (NC).

As one of the provincial members of the Central China Power Grid, Hubei produces electricity as a supplier, while in the meantime, it is an electricity purchaser from other power grids. Such trans-regional and inter-provincial electric power transmissions in-between these State Grids (as illustrated in Figure 1) balance the nationwide supply-and-demand [24]. Therefore, this increases the complexity and difficulty of the actual value of Hubei consumptive-electricity carbon intensity.

According to China Electric Power Yearbook 2018, the percentages of the annual consumptive electricity in Hubei in 2017 were 96.98% self-production, 1.10% net-import from North China Power Grid, 1.13% net-import from Northeast China Power Grid, 0.79% mixed-import of hydropower from Tibet, Southwest and Hunan [21]. We use these proportions and the CO_2_ emission factors, 751 kg/MWh for Hubei (Central China), 1066 kg/MWh for North China [25], 58 kg/MWh for Northwest renewables and 13 kg/MWh (Table 3) for the average hydropower from Tibet, Southwest China and Hunan, for the calculation of the consumptive-electricity carbon footprints (CFs) during the COVID-19 lockdown. The equivalent carbon intensity of Hubei consumptive electric power via the means of weighted summation is calculated to be 0.844 kg/kWh.

Having the emission factor decided, the monthly CF is calculated by the following equation:

(6) $$CF_{mon.}=E_{cons.}\times EF_{eq.}=E_{cons.}\times \overset{n}{\underset{i=1}{\sum }}\left(r_{i}\times EF_{i}\right)\;$$

where *i* is the index of power grid; *n* represents the number of associated power grids; $CF_{mon.}$ is the monthly CF of consumptive electricity; $E_{cons.}$ is the amount of monthly consumed electric power; $EF_{eq.}$ is the equivalent carbon emission factor of Hubei consumptive electricity; $r_{i}$ indicates the percentage of electricity purchased from *i*th power grid; $EF_{i}$ is the carbon emission factor of electricity generation from *i*th power grid.

### 2.4. Test of Uncertainties

Uncertainties mainly come from the propagation of data errors, inaccurate modeling, incomplete system components inclusion, and numerical truncations, etc. In practice, the uncertainties could be tested by either analytical methods or random sampling approaches. Here in this study, we use the Monte Carlo (MC) sampling method to test the effect of error propagation and probability distribution of the outputs. The MC method is a commonly used technique for uncertainty analysis and is expressed below [30,31].

(7) $$\eta =\eta \left(x_{1},x_{2},\ldots x_{M}\right)\;$$

(8) $$\eta_{i}=\eta \left(x_{1i},x_{2i},\ldots x_{Mi}\right)\;$$

(9) $$\bar{\eta }=\frac{1}{N}\overset{N}{\underset{i=1}{\sum }}\eta_{i}\;$$

(10) $$\sigma^{2}=\frac{1}{N-1}\overset{N}{\underset{i=1}{\sum }}{\left(\eta_{i}-\bar{\eta }\right)}^{2}\;$$

where (*x*_1*i*_, *x*_2*i*_, … *x_Mi_*) is the *i*th random sampling of input *x*_1_, *x*_2_, … *x_M_*; *η* stands for an arbitrary output, which is dependent on *x*_1_, *x*_2_, … *x*_M_; *η_i_* is the output parameter decided by the *i*th random sampling; $\bar{\eta }$ represents the mean output of parameter *η* after *N* times of random samplings; *σ* is the standard deviation of output *η*.

As for the input uncertainties, we assume the following purchasing scenarios for the consumptive electricity: (1) Scenario I: all consumptive electricity was self-produced within the Central China Power Grid; (2) Scenario II: Imported electricity was all from North China Power Grid, which has the largest CO_2_ emission factor; (3) Scenario III: Imported electricity was all generated from hydropower, which has the lowest CO_2_ emission factor.

We calculate the equivalent emission factors for the three scenarios and use the maximum and minimum as the upper and lower limits for the uncertainty range of the emission factor. For the monthly statistical electricity consumption, we assume there were ±5% errors. 10,000 samplings were conducted for the calculation of the monthly CF changes. The reliabilities of our results are tested by the uncertainty analysis results through the MC method.

## 3. Results

### 3.1. Changes of Electricity-Generation CO_2_ Emissions

**Figure 2:**
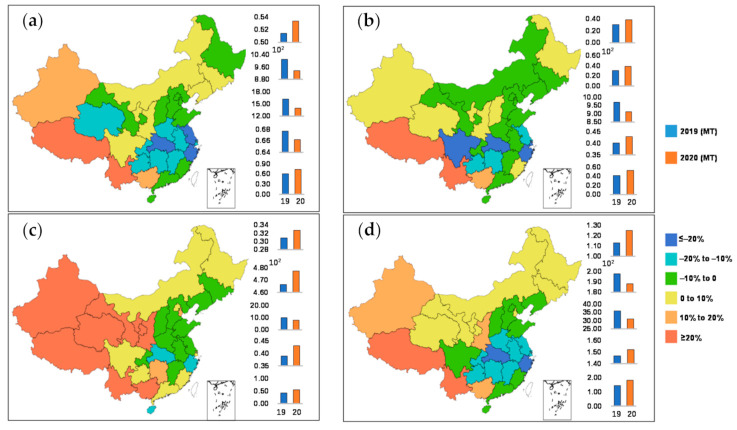
Changes in electricity-generation CO_2_ emissions in Mainland China. (**a**) Total changes during January–April 2020; (**b**) changes in January–February 2020; (**c**) changes in March 2020; (**d**) changes in April 2020.

Our bottom-up analysis results show that the electricity-generation-caused CO_2_ emissions fell sharply after the Wuhan travel ban was initiated on 23 January 2020. Considering various provincial electricity-generation carbon burdens due to different electricity generation mix [28,32,33], Figure 2 compares the nationwide distribution of electricity-generation CO_2_ emission changes during the COVID-19 lockdown period in comparison with the same months in the year prior.

Our calculation reveals a 4.9% reduction in national electricity-generation CO_2_ emissions during January–April 2020 (Table 4). The biggest declines of the four-month total were observed in ECG, with a reduction of 15.0% during the four months, followed by CCG, 13.8%. During January–February, decrease of carbon emissions took place in four out of six China national power grids, with the largest by 20.6% in ECG, followed by 16.8% in CCG, 4.6% in NCG, and 3.3% in SCG. The monthly reductions of ECG were 20.6%, 13.7% and 5.0% during January–February, March and April, and 16.8%, 16.8% and 3.1% for CCG. This could be explained by the fact that these two grids covered provinces which are more populated and more industrialized compared to the rest of Mainland China. The carbon emissions from electricity generation in NWCG maintained an increase in January–April 2020, which is likely due to the provinces being less affected by the COVID-19 pandemic, and so therefore the lockdown levels were not as strict as other national grid covered provinces to lower the power generation. At the province level, the top three CO_2_ emission declines were observed in Hubei, the most COVID-19 affected province, followed by Zhejiang and Jiangsu, with their GDPs ranked the second and the fourth in China. Since the lockdown measure was implemented on 23 January 2020, the total Hubei provincial electric-power demand and consumption decreased steeply as a consequence. Hubei, as the most affected province, witnessed declines of electricity-generation CO_2_ emissions of 25.5% in January–February, 42.7% in March and 27.3% in April. As shown in Table 4, the most considerable change was observed in February 2020, with a 34.65% decrease lower than the same month in 2019.

Our results found out reduction of electricity form coal contributed 951 Mt out of 994 Mt (Table 4), nearly 95.7% of the overall national CO_2_ emission reduction during the four months, because coal has been the main source of China’s electricity, accounting for 74.5% and 73.7% of the total electricity productions in January–April 2019 and 2020, and thus the most significant CO_2_ emission contributor. Hydro power contributed 4.9% of the total reduction, with an amount of 48 Mt CO_2_ emission. Considering their low-carbon advantage, the contributions to total CO_2_ emission reduction from other sources were limited, due to their lesser percentage in China’s power generation mix.

**Table 4:** China nationwide electricity-generation CO_2_ changes in January–April 2020 compared with same months in 2019.

Months	Generation (10^8^ kWh)		CO_2_ Emissions (Mt)		Change (%)
	2019	2020	2019	2020	
January– April	22,120	21,284	20,166	19,172	−4.9
	1256	1389	11	13	10.6
	16,472	15,680	19,767	18,816	−4.8
	2990	2587	359	310	−13.5
	1048	1087	15	15	3.7
	353	443	14	18	25.5
January– February	572	593	5	5	3.8
	8427	7807	10,112	9368	−7.4
	1352	1164	162	140	−13.9
	484	473	7	7	0.0
	147	179	6	7	21.5
	10,982	10,216	10,292	9527	−7.4
March	342	432	3	4	26.4
	4160	3894	4991	4673	−6.4
	809	761	97	91	−5.9
	287	306	4	4	0.0
	101	129	4	5	27.3
	5698	5525	5100	4778	−6.3
April	343	364	3	3	6.1
	3886	3979	4663	4775	2.4
	829	662	99	79	−20.1
	278	308	4	4	0.0
	104	135	4	6	29.6

### 3.2. Changes of Consumptive-Electricity Carbon Footprints

**Figure 3:**
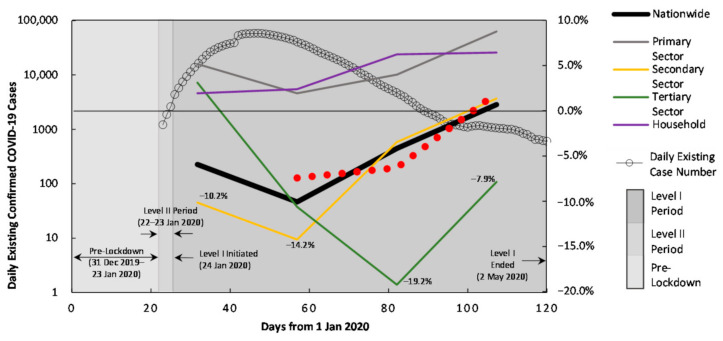
Changes of consumptive-electricity related CO_2_ emissions in January–April 2020. The daily existing confirmed COVID-19 cases in Mainland China is included.

Figure 3 plots the curves from January to April 2020 for daily existing confirmed COVID-19 cases [34], the monthly electric-power consumption and generation changes. When the confirmed case number soared in February, the biggest drops occurred correspondingly on both generation and consumption sides, with a total decrease of 10.1% for nationwide consumption and an average 7.4% for nationwide generation (Table 5). From the perspective of individual economy sectors consuming electricity, the secondary sector saw the most drastic reduction of 14.2% in February, which can be explained by the fact that the lockdown measures required industrial businesses stay closed till 11 March. The primary and residential household sectors did not witness as big changes as the secondary and tertiary sectors in electricity consumption, which is likely due to the fact that the agricultural activities are usually not as active during the Chinese Spring Festival season, and that the household electric consumption per capita does not differ much no matter whether people lived with or without the constrains of lockdown measures.

Our province-wide data acquisition process ended up with only 8 provinces’ statistics being available, include Shanghai, Jilin, Henan, Hunan, Anhui, Guizhou, Qinghai and Hubei. Detailed analysis of Hubei is presented in the case study of Hubei. As shown in Among the 7 provinces excluding Hubei, 5 of them witnessed outstanding declines in electric power consumptions from January to March 2020, except for Jilin and Qinghai, which were less affected by COVID-19 during this period of time.

**Table 5:** Consumptive carbon footprint changes (%) in January–April 2020, compared with same month of 2019.

Sectors	Generation (10^8^ kWh)		CO_2_ Emissions	
Nationwide	−5.9%	−10.1%	−4.2%	0.7%
Primary Sector	5.2%	1.9%	4.0%	8.8%
Secondary Sector	−10.2%	−14.2%	−3.5%	1.3%
Tertiary Sector	3.1%	−10.6%	−19.2%	−7.9%
Household	1.9%	2.4%	6.2%	6.4%
Generation	-	−7.4%	−6.3%	1.9%

The secondary sectors of these provinces mostly experienced apparent reduction of power demand due to social distancing protocol caused short of labor and associated with production slowing down. The most significant declines of power consumption of these provinces occurred in March, when people were not able to go back to work as usual; thus, the related services had to remain closed. Similar as the nationwide case, the primary and residential sectors did not see drops as massive as in the secondary and tertiary sectors. However, as a province neighboring Hubei, Henan’s electricity consumption and its related indirect carbon emissions increased by 113.3% in March 2020, compared to the same month of the year prior. This is most likely because of the switching of the residents’ life pattern from business-as-usual to stay-home after the Spring Festival, which ended up with the household power demand soaring during their daily at home activities.

Our analyses show that the nationwide electricity generation and consumption did not coincide with each other as expected according to their change curves, although they both plummeted after the Chinese government initiated the lockdown policies. The average decline of the nationwide electricity consumption was approximately 8%, yet the decline on the generation side was 7.4%, which is approximately a difference of as much as 6 Mt CO_2_ emissions.

### 3.3. Results of Hubei Case

#### 3.3.1. Changes of Hubei Monthly Consumptive-Electricity CFs

**Figure 4:**
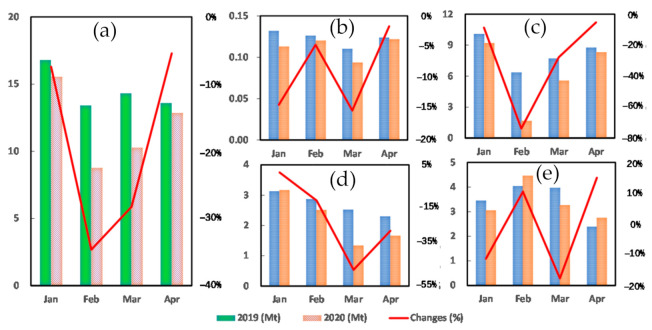
Changes in Hubei consumptive electric power CF from January through April in 2020, compared to the baseline year, 2019. (**a**) Provincial; (**b**) Primary Sector; (**c**) Secondary Sector; (**d**) Tertiary Sector; (**e**) Residential.

Since the first lockdown measure was implemented on 23 January 2020, the total Hubei provincial electric power demand and consumption decreased steeply as a consequence (Figure 4). As shown in Table 6, the most considerable change was observed in February 2020, with a 34.65% decrease below the same month in 2019 (Figure 4a). The associated with CF reduction of the consumptive electricity was 4.65 Mt CO_2_. The secondary sector was the most contributing sector to the CF reduction with a reduction in electricity consumption of 5573 GWh in March 2020 below the same month in 2019, which resulted in a CF reduction of 4.70 Mt CO_2_.

#### 3.3.2. Primary Sector of Hubei

As shown in Figure 4b, the consumption of electric power in the primary sector was an order of magnitude less compared to the secondary and tertiary sectors, as well as the residential. The monthly changes of the consumptive-electricity CF in January through April in 2020 were −0.019 Mt CO_2_, −0.006 Mt CO_2_, −0.017 CO_2_ and −0.002 Mt CO_2_, which contribute percentage reductions of 14.34%, 4.67%; 15.24% and 1.69% below the same months in 2019, respectively. We notice that 25 January 2020 was the Chinese Spring Festival, the lockdown measures restricted the labor transition between cities and rural areas, and this caused the electricity demand decrease in the agricultural work. Most of February 2020, from 1st through 22 February 2020 fell on the first Chinese lunar month, which is the most important timing for social networking such as visiting and gathering with relatives and friends, thus relatively not as productive a period of time. Therefore, February 2020 saw neither a sharp reduction of electricity consumption nor its CF. Detailed calculation results is shown in Table 6, Table 7, Table 8 and Table 9.

#### 3.3.3. Secondary Sector of Hubei

The main subsectors of the secondary sector are industry and construction. The changes of sectoral consumptive-electricity CFs in the first four months in 2020 were calculated to be −0.88 Mt CO_2_, −4.70 Mt CO_2_, −2.14 Mt CO_2_ and −0.46 Mt CO_2_. The corresponding percentage reductions were 8.69%, 73.9%, 27.81% and 5.22%. As shown in Figure 4c, the most significant reduction of industrial subsector was observed in February, which was 75.03%, and this is due to the lockdown measure requiring that industrial businesses stay closed till 11 March. Whereas the sharpest change in construction subsector, a decrement of 70.70% happened in March 2020, which was because of the shortage of construction labor, whose mobilization back to work was restricted by the lockdown measures, after majority of them went back to their hometown in rural areas for holidays.

#### 3.3.4. Tertiary Sector of Hubei

The three biggest electricity consuming subsectors of the tertiary sector, according to data of 2019, were public service and management, whole sales and retails, and transportation, storage and logistics [35]. As shown in Figure 4d, decreases of their electric power consumption caused CFs were 39.07%, 62.05% and 53.05%, and all of them happened in March 2020. This could be explained by their dependence on transportation and human to human interactions. Conversely, the information service subsector witnessed increments of 11.96%, 14.17%, 10.87% and 13.91% from January through April 2020. This was due to the increase of internet usage as people spent more time working, shopping, and entertaining online from home compared to their normal daily workday lives.

#### 3.3.5. Residential Sector of Hubei

Changes of this sectoral electricity consumption and its CFs in the first four months of 2020 were −11.27%, 10.52%, −17.69% and 15.05%, as shown in Figure 4e. In order to understand this, we take apart the changes in two subsectors, urban and rural, month by month. Nothings went abnormally in January 2020 until the COVID-19 lockdown was implemented on 23 January. These measures were the main contributing factor as they cause the shutdown of social behaviors. February saw an increment of 3.01% for urban residential electricity consumption and its CF, however 26.60% for rural. Most rural migrant workers were required not to go back to work and stay home and they were the main consumers of the increased household electric power. From 11 March, Hubei started to recover its public transportation, business running, rural migrant workers returning and inter-provincial people mobilizing etc. [36] Hence, the households’ electricity consumption of March decreased by 17.69% due to shortening stay at home. In April, urban electricity consumption and CF became close to same month of 2019, as the residential life and production activities was getting normalization as usual. However, a 44.93% increase was observed for rural subsector, and as per our observation, this was mainly due to the fact that majority of the individual business owners from rural areas, such as food and snack vends owners, were restricted from getting back to cities. Hence, they contributed to the increment of the rural household consumptive-electricity CFs.

The scope of our analysis simplifies the complexity of the electric power supply mix in Hubei, as well as the variations of technological and regional electricity-production carbon intensity therein, and thus increases the uncertainties of the analysis results. Also, we realize that the official statistics would’ve possibly brought inherent errors that damage the reliability of our analysis. As per these practical concerns, we conducted an MC uncertainty analysis to test the input-output uncertainty propagation and confidence level of our calculation results. We also recognized the seasonal factors may affect the rationality of our study, as it is a commonly known consensus and thus we compared the same monthly data of 2020 and 2019 to avoid the bias that may be possibly raised. We did not include greenhouse gases other than CO_2_, which would also bring uncertainties to our analysis results, and this should be addressed in future study to make more sense of our work.

### 3.4. Uncertainties

There have been uncertainties regarding China’s energy statistics, which raises a huge challenge for bottom-up studies relying on these statistical data. Considering that coal is the main CO_2_ source in electricity sector [37,38], we used the estimated apparent uncertainty of coal consumption (14%) and calculated with an interpretation method based on the reported data of 1996–2003, 2004–2012, and 2013 [39]. Our 10,000 runs of sampling returned results for electricity-production CO_2_ emission reductions of January-February, March and April are −73.89, −31.65 and 11.03 Mt (means) and 39.86, 19.87 and 19.24 Mt (standard deviations).

**Figure 5:**
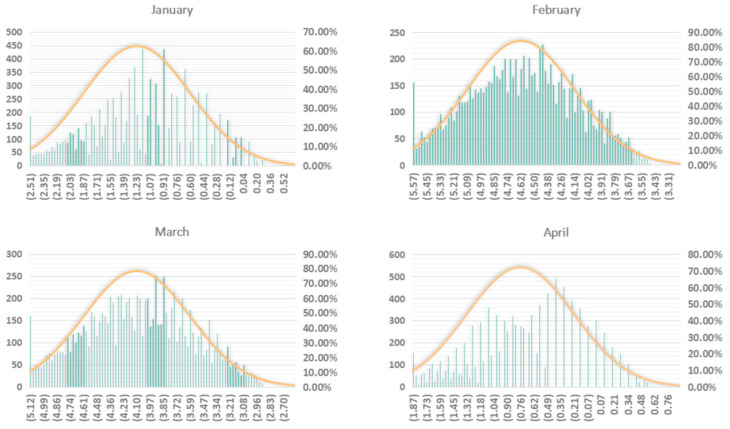
Probability distributions of the monthly CF changes of Hubei consumptive electric power from January through April in 2020, compared to the baseline year, 2019.

As for case of Hubei, we used the data of 2017 to calculate the percentages of purchased electricity from other power grids. As shown in Figure 5, The means of the CF changes from January through April, following 10,000 samplings are calculated to be −1.23, −4.62, −4.10 and −0.76 Mt CO_2_, and their standard deviations are 0.64, 0.47, 0.51, and 0.55 Mt CO_2_. The confidences of our calculated monthly CF changes of Hubei consumptive electricity are 62.50%, 83.91%, 78.01% and 72.14%.

## 4. Discussion and Conclusions

While our study is limited to China due to data availability, it reflects an overlook of the COVID-19 impacts on the nationwide electricity-carbon nexus, exploring spatial, economic sectoral, as well as technological dimensions, and examining both the generation and consumption sides. The results show that the lock-down measures caused dramatic carbon footprint changes at both nationwide and provincial levels in China. We believe that if adopted, our method and results could be inspirational for similar analysis and diagnostics in other geographical areas.

### 4.1. Implications

Our study should raise awareness for decision makers in charge of China’s overall environmental management and sustainability development. We therefore conclude with the following recommendations for post-COVID planning:

- providing potential action plans, such as curbing large-scale electricity production activities, at national and provincial levels;
- switching ways of some of the business behavior (e.g., from offline to online);
- avoiding inconsistency within the governmental statistical data, as our study shows that some of the consumptions exceeded the electricity generation of the same period, which could result from a lack of sufficient communication and cross-check between different parties providing the two sets of data, and thus, more efforts should be spent on data validation;
- revising governmental statistical database;
- making up the missing provincial electricity consumption data, as data of some provinces are not available on their governmental statistical websites, which renders a more detailed analysis challenging;
- updating statistical data in a more timely and accountable manner.

### 4.2. Limitations and Future Study

The main limitations of our study are:

- This study only focuses on the most COVID-19 affected period from January to April 2020, and a thorough study on the post-COVID-19 impacts is beyond our work, and this should be further investigated in our future work. This would reveal whether the impacts are temporary or the effects are long-term.
- The reliability of our study results needs to be further improved, since the data quality should be improved as well, as discussed in the above section.
- The lock-down measure conduction levels differed from province to province, and this should be taken into account in our future study.

Overall, our study design showcases an innovative and systematic approach by integrating a thorough uncertainty analysis, which could be used as a reference to measure the impacts of COVID-19 on other utilities. Our study design could also be applicable for countries or areas having practiced social distancing policies and measures, which could help provide insights into their overall environmental management or decarbonization.

## Figures and Tables

**Table 1:** Geographical boundaries of six regional power grids within China.

Regional Power Grids	Covered Provincial Areas
North China	Beijing, Tianjin, Hebei, Shanxi, Shandong, West Inner Mongolia
Northeast China	Liaoning, Jilin, Heilongjiang, East Inner Mongolia
East China	Shanghai, Jiangsu, Zhejiang, Anhui, Fujian
Central China	Jiangxi, Henan, Hubei, Hunan, Chongqing, Sichuan
Northwest	Shaanxi, Gansu, Ningxia, Qinghai, Xinjiang
South China	Guangdong, Guangxi, Yunnan, Guizhou, Hainan

Data adapted from [25,26].

**Table 2:** GHG Burden for China’s National and Regional Grids.

Grids	North	Northeast	Northwest	Central	East	South
Carbon Intensity (gCO_2_/kWh)	1066	1014	833	751	836	569

Data adapted from [27].

**Table 3:** China’s electricity CO_2_ emission intensities of renewables.

Sources	Renewables	Hydro
Carbon intensities (kg CO_2_ /MWh)	58	13

Data adapted from [28,29].

**Table 6:** Hubei sectoral carbon footprint changes of January 2020 compared to January 2019.

Sectors	Mar 2019	Mar 2020	Change	Carbon Footprint (Mt)	%
Total Provincial	198.97	184.23	−14.74	−1.24	−7.41
Primary Sector	1.56	1.34	−0.22	−18.91	−14.34
Secondary Sector	119.46	109.08	−10.38	−0.88	−8.69
Industry	115.79	106.71	−9.08	−0.77	−7.84
Manufacturing of Oil, Coal, and Other Fuels	2.41	-	-	-	-
Manufacturing of Chemical Materials and Products	15.98	-	-	-	-
Manufacturing of Non-metallic Products	12.02	-	-	-	-
Ferrous Metal Refining & Rolling	15.83	-	-	-	-
Nonferrous Metal Refining & Rolling	5.71	-	-	-	-
Electric and Thermal Power Production and Supply	21.67	-	-	-	-
Construction	4.22	3.37	−0.85	−71.91	−20.20
Tertiary Sector	37.11	37.58	0.47	39.77	1.27
Transportation, Storage and Logistics	5.79	5.56	−0.23	−19.28	−3.95
Information communication, Software and Information Technological Service	1.64	1.84	0.20	16.58	11.96
Wholesales and Retails	7.56	7.98	0.42	35.81	5.61
Accommodation and Catering	2.92	3.04	0.12	9.95	4.03
Finance	0.49	0.55	0.06	4.87	11.70
Real Estate	4.84	5.15	0.31	26.38	6.46
Rental and Business Service	0.72	0.65	−0.07	−5.80	−9.55
Public Service and Management	11.87	11.07	−0.80	−67.62	−6.75
Urban and Rural Residential Households	40.84	36.23	−4.60	−388.60	−11.27
Urban Residential Households	28.65	24.52	−4.13	−348.65	−14.42
Rural Residential Households	12.19	11.71	−0.47	−39.95	−3.88

Data adapted from [35].

**Table 7:** Hubei sectoral carbon footprint changes of February 2020 compared to February 2019.

Sectors	Mar 2019	Mar 2020	Change	CF (Mt)	%
Total Provincial	158.86	103.82	−55.04	−4.65	−34.65
Primary Sector	1.50	1.43	−0.07	−5.90	−4.67
Secondary Sector	75.41	19.68	−55.73	−4.70	−73.90
Industry	73.17	18.27	−54.90	−4.63	−75.03
Manufacturing of Oil, Coal, and Other Fuels	2.04	-	-	-	-
Manufacturing of Chemical Materials and Products	12.53	-	-	-	-
Manufacturing of Non-metallic Products	5.08	-	-	-	-
Ferrous Metal Refining & Rolling	12.71	-	-	-	-
Nonferrous Metal Refining & Rolling	5.05	-	-	-	-
Electric and Thermal Power Production and Supply	8.57	-	-	-	-
Construction	2.59	1.94	−0.66	−55.49	−25.34
Tertiary Sector	34.06	29.78	−4.28	−361.59	−12.58
Transportation, Storage and Logistics	5.77	4.74	−1.03	−86.57	−17.79
Information communication, Software and Information Technological Service	1.64	1.87	0.23	19.63	14.17
Wholesales and Retails	7.24	5.67	−1.56	−132.03	−21.61
Accommodation and Catering	2.97	2.27	−0.70	−59.26	−23.63
Finance	0.56	0.53	−0.03	−2.18	−4.61
Real Estate	4.34	3.74	−0.60	−50.78	−13.87
Rental and Business Service	0.61	0.49	−0.11	−9.65	−18.85
Public Service and Management	9.90	9.25	−0.65	−54.88	−6.57
Urban and Rural Residential Households	47.90	52.93	5.04	425.29	10.52
Urban Residential Households	32.65	33.64	0.98	83.08	3.01
Rural Residential Households	15.24	19.29	4.05	342.12	26.60

Data adapted from [35].

**Table 8:** Hubei sectoral carbon footprint changes of March 2020 compared to March 2019.

Sectors	Mar 2019	Mar 2020	Change	CF (Mt)	%
Total Provincial	169.63	121.67	−47.95	−4.05	−28.27
Primary Sector	1.31	1.11	−0.20	−16.83	−15.24
Secondary Sector	91.35	65.95	−25.40	−2.14	−27.81
Industry	88.99	65.23	−23.76	−2.01	−26.70
Manufacturing of Oil, Coal, and Other Fuels	1.82	1.92	0.10	8.33	5.42
Manufacturing of Chemical Materials and Products	11.63	10.58	−1.05	−88.63	−9.03
Manufacturing of Non-metallic Products	8.35	2.07	−6.28	−0.53	−75.17
Ferrous Metal Refining & Rolling	14.22	12.94	−1.28	−108.31	−9.02
Nonferrous Metal Refining & Rolling	5.14	4.10	−1.05	−88.23	−20.33
Electric and Thermal Power Production and Supply	13.07	18.83	5.76	486.35	44.09
Construction	2.82	0.83	−1.99	−168.36	−70.70
Tertiary Sector	29.87	15.85	−14.02	−1.18	−46.93
Transportation, Storage and Logistics	5.02	2.36	−2.66	−224.77	−53.05
Information communication, Software and Information Technological Service	1.51	1.67	0.16	13.82	10.87
Wholesales and Retails	6.07	2.31	−3.77	−318.13	−62.05
Accommodation and Catering	2.29	0.91	−1.38	−116.29	−60.12
Finance	0.48	0.35	−0.13	−10.95	−26.81
Real Estate	4.00	1.92	−2.08	−175.79	−52.04
Rental and Business Service	0.54	0.23	−0.31	−25.84	−56.95
Public Service and Management	8.88	5.41	−3.47	−292.80	−39.07
Urban and Rural Residential Households	47.09	38.76	−8.33	−703.11	−17.69
Urban Residential Households	29.65	21.75	−7.89	−0.67	−26.62
Rural Residential Households	17.45	17.01	−0.44	−37.09	−2.52

Data adapted from [35].

**Table 9:** Hubei sectoral carbon footprint changes of April 2020 compared to April 2019.

Sectors	Mar 2019	Mar 2020	Change	CF (Mt)	%
Total Provincial	161.03	152.31	−8.72	−0.74	−5.42
Primary Sector	1.47	1.44	−0.02	−2.09	−1.69
Secondary Sector	103.91	98.49	−5.42	−0.46	−5.22
Industry	101.28	97.30	−3.99	−0.34	−3.94
Manufacturing of Oil, Coal, and Other Fuels	2.00	1.83	−0.17	−14.55	−8.60
Manufacturing of Chemical Materials and Products	13.19	15.92	2.74	230.91	20.75
Manufacturing of Non-metallic Products	12.20	8.50	−3.69	−311.53	−30.27
Ferrous Metal Refining & Rolling	16.09	15.36	−0.73	−61.88	−4.56
Nonferrous Metal Refining & Rolling	4.89	4.40	−0.49	−41.08	−9.96
Electric and Thermal Power Production and Supply	12.60	14.80	2.20	185.43	17.43
Construction	3.13	1.67	−1.46	−123.17	−46.57
Tertiary Sector	27.32	19.78	−7.54	−636.52	−27.61
Transportation, Storage and Logistics	5.27	3.53	−1.74	−147.05	−33.07
Information communication, Software and Information Technological Service	1.60	1.82	0.22	18.80	13.91
Wholesales and Retails	5.61	3.47	−2.14	−180.71	−38.19
Accommodation and Catering	1.81	0.97	−0.84	−70.83	−46.32
Finance	0.42	0.40	−0.02	−1.63	−4.63
Real Estate	3.40	2.20	−1.20	−101.63	−35.38
Rental and Business Service	0.45	0.35	−0.10	−8.33	−21.92
Public Service and Management	7.43	5.62	−1.80	−152.27	−24.29
Urban and Rural Residential Households	28.34	32.60	4.26	359.85	15.05
Urban Residential Households	18.34	18.11	−0.23	−19.38	−1.25
Rural Residential Households	10.00	14.49	4.49	379.22	44.93

Data adapted from [35].

## Data Availability

The data that support the findings of this study are available from the corresponding author, upon reasonable request.
